# The Use of Positron Emission Tomography in Soft Tissue Sarcoma Patients under Therapy with Trabectedin

**DOI:** 10.3390/md7030331

**Published:** 2009-07-17

**Authors:** Bernd Kasper, Thomas Schmitt, Patrick Wuchter, Antonia Dimitrakopoulou-Strauss, Anthony D. Ho, Gerlinde Egerer

**Affiliations:** 1 Department of Internal Medicine V, University of Heidelberg, Im Neuenheimer Feld 410, D-69120 Heidelberg, Germany; E-Mails:Thomas.Schmitt@med.uni-heidelberg.de (T.S.);Patrick.Wuchter@med.uni-heidelberg.de (P.W.);Anthony.Ho@med.uni-heidelberg.de (A.H.);Gerlinde.Egerer@med.uni-heidelberg.de (G.E.); 2 Clinical Cooperation Unit Nuclear Medicine, German Cancer Research Center, Im Neuenheimer Feld 280, D-69120 Heidelberg, Germany; E-Mail:ads@ads-lgs.de

**Keywords:** fluorodeoxyglucose, positron emission tomography, RECIST, soft tissue sarcoma, trabectedin

## Abstract

**Background:**

We used 2-deoxy-2-[^18^F] fluoro-D-glucose (FDG) positron emission tomography (PET) to evaluate the FDG uptake in patients with advanced and/or metastatic soft tissue sarcoma (STS) undergoing therapy with Ecteinascidin-743 (ET-743, Trabectedin, Yondelis™).

**Patients and Methods:**

The pilot study included nine patients with metastatic STS receiving a minimum of one cycle of treatment with trabectedin. Patients were examined using PET prior to onset of therapy and after completion of one or three cycles of trabectedin. Restaging according to Response Evaluation Criteria in Solid Tumours (RECIST) was performed in parallel using computed tomography (CT) and/or magnetic resonance imaging (MRI) and served for reference.

**Results:**

Clinical outcome of nine evaluable patients was as follows: one patient with partial remission (PR), three patients with stable disease (SD), and five patients with progressive disease (PD). A more than 40% decrease of the standardized uptake value (SUV) of sequential PET examination could be demonstrated for the responding patient (PR), whereas patients with SD or PD showed a stable SUV, but no increase in SUV.

**Conclusion:**

To our knowledge, this is the first small series of patients being treated with trabectedin and monitored using sequential PET imaging demonstrating SUV stabilization in nearly all monitored patients.

## 1. Introduction

Soft tissue sarcomas (STS) are a heterogeneous group of connective tissue malignancies arising from tissue of mesenchymal origin. They constitute less than 1% of all adult malignancies. The five-year overall survival rate in patients with STS of all stages amounts to only 50–60% [1]; most patients die of metastatic disease, which becomes evident in 80% of the cases within two to three years after initial diagnosis [2]. Despite improvements in local tumour control rates, the treatment of patients with high risk STS remains challenging. For patients with no evidence of metastatic disease surgery is the primary treatment of choice. The high rate of distant disease recurrence suggests that undetectable metastatic disease is present in a significant percentage of patients with large, high grade STS at the time of surgery. Therefore an effective systemic treatment with chemotherapy is needed. Doxorubicin and ifosfamide are the most active single-agents in the therapy of STS with response rates above 20% [3]. So far, the benefit of neither adjuvant chemotherapy [4] nor neoadjuvant chemotherapy has yet been finally clarified [5].

Therefore, it is important to identify patients who are likely to benefit from chemotherapy or other molecular targeted agents. Morphologic imaging modalities like CT and MRI can be used for the assessment of tumour localization, size, and infiltration of the surrounding tissue as well as presence of satellite metastases. However, it has not been well established whether a significant change in the tumour size is a meaningful tool of outcome of patients with STS. Standard radiographic response has not correlated consistently with histological response or with disease-free or overall survival [6–7]. Other methods to identify patients who are likely to benefit from chemotherapy or other agents would be useful. Therefore, PET with ^18^F-FDG has found increasing use in oncology, because it allows functional imaging of viable tumour tissue [8]. However, not all tumours are PET avid, despite being viable.

It was shown that FDG PET can visualize STS and detect local and distant recurrence of disease [9–10]. It was demonstrated that the SUV correlates well with the metabolic rate of FDG accumulation in tumour cells [11]. Hence, the SUV could function as an easily measurable surrogate of tumour viability during therapy. For other tumour entities, correlation between FDG accumulation after neoadjuvant chemotherapy and histological response as well as tumour response was shown, for example in breast cancer [12], oesophageal cancer [13], colorectal cancer [14], and gastrointestinal stromal tumours [15]. In a group of 46 patients with localized, intermediate/high grade, extremity STS, Schuetze et al. could demonstrate that changes of the SUV before and after neoadjuvant chemotherapy can be used to predict therapy outcome [16]. The multivariate analysis found a correlation between lack of response and increased risk of disease recurrence, metastases and death after appropriate local control of sarcoma. This study suggested that FDG PET can act as a non-invasive method in high risk extremity STS to predict patients who are less likely to benefit from doxorubicin-based chemotherapy [17].

Ecteinascidin-743 (ET-743, Trabectedin, Yondelis™) is a tetrahydroisoquinoline alkaloid isolated from the Caribbean marine tunicate, *Ecteinascadia turbinate*. ET-743 is a DNA guanine-specific minor groove binding agent that blocks the cell cycle in the late S and G phase. Five phase II trials in Europe and the United States revealed a response rate of 10% in pre-treated and 18% in untreated patients with response duration of about ten months and a progression-free survival of 24% at 6 months and 9% at 12 months [18–19]. In particular, anti-tumour activity has been demonstrated in leiomyosarcomas and liposarcomas. ET-743 seems to be especially active in the myxoid variant of liposarcomas associated with translocations of t(12;16)(q13;p11) or t(12;12)(q13;q12); among 15 retrospectively reviewed cases eight objective responses were found [20]. There is only one phase I study monitoring patients being treated with trabectedin with FDG PET. Although there were no objective responses to therapy, clear evidence of anti-tumour activity was observed in a patient with epithelioid mesothelioma as confirmed by PET [21].

The purpose of the present study, which is the first covering this topic, was to analyse and discuss semi-quantitative FDG PET measurements in STS patients treated with trabectedin.

## 2. Patients and Methods

### Patients

The pilot study includes nine patients with STS. Patients’ characteristics including gender, age, histologies and grading according to the Federation Nationale des Centres de Lutte Contre le Cancer (FNCLCC) system [22], tumour and metastatic sites as well as line of treatment are summarized in Table 1. All patients were referred to our outpatient service with a diagnosis of a STS confirmed by histology obtained from surgical specimens. Tumour specimens were classified according to the FNCLCC system. Patients were treated with trabectedin in the Department of Internal Medicine V, University of Heidelberg between September 2008 and June 2009. The research was carried out according to the principles set out in the Declaration of Helsinki in 1964 and all subsequent revisions.

### Ecteinascidin-743

Nine patients with metastatic disease received trabectedin in a dose of 1.5 mg/m^2^/day 1 as a 24 hour continuous infusion, repetition day 22 for at least one cycle. Altogether, we performed 18 cycles of therapy; treatment was continued until disease progression. One patient received trabectedin in a reduced dose of 1.3 mg/m^2^ because of liver metastases with elevated liver parameters. In spite of CTCAE grade I/II fatigue, nausea and neutropenia, no major (grade III and IV) toxicities occurred.

### Imaging studies

Patients were examined using FDG PET prior to onset of therapy and after completion of one to three cycles of trabectedin. Conventional imaging of the same target lesion using CT and/or MRI was performed in parallel to determine the response according to RECIST criteria. This data served as reference to evaluate the response determined with FDG PET. Dynamic PET studies were performed after intravenous injection of 300–370 MBq FDG for 60 min. A dedicated PET system (ECAT EXACT HR plus, Siemens, Erlangen, Germany) was used for patient studies as described before [23]. The last images (55–60 minutes post-injection) were used for semi-quantitative analysis. PET cross-sections were reconstructed with an image matrix of 256 × 256 using an iterative reconstruction program. Images were scatter- and attenuation-corrected. Volumes of interest (VOI) were placed over the lesion. To acquire information about the tumour viability, the hyper-metabolic areas of the tumours were evaluated and hypo-metabolic areas that correlate to necrotic tissue were excluded. The SUV in the tumour was calculated according to the following equation: SUV = tissue concentration (MBq/g)/[injected dose (MBq)/body weight (g)]. The SUV reflected the average SUV value provided by the quantification software in a VOI. This value is more robust than the maximum SUV (SUV_max_), because it is less influenced by the parameters used for the image reconstruction as well as by potential artefacts. The analysis of the PET images was performed together by two nuclear medicine physicians using the software package Pmod (PMod Technologies Ltd., Adlisvil, Switzerland) [24].

### Statistical analysis

Due to the small number of patients we performed a descriptive analysis of the data. Progression-free survival was defined as the time interval from the date of therapy induction until the tumour recurred or the patient died of sarcoma-related causes.

## 3. Results and Discussion

### Clinical response based on RECIST criteria

First CT and/or MRI scan was performed in all patients prior to onset of therapy with trabectedin. Restaging was performed using CT and/or MRI after one (n = 2) or three (n = 4) cycles of trabectedin. The remission status was evaluated according to RECIST criteria based on the tumour shrinkage in the CT and/or MRI scan. Clinical outcome according to RECIST criteria was as follows: one patient with PR, three patients with SD, and five patients with PD. The median progression-free survival from the date of therapy induction for all patients was 2.8 months [range: 0–7] with a median follow-up of 3.8 months [range: 1–7].

### Clinical response based on PET imaging

The median average SUV prior to onset of targeted therapy with trabectedin was 7.6 [range: 1.2–19.3] in comparison to 5.8 [range: 1.4–20.1] after treatment. The median SUV_max_ was 14.1 [range: 4.5–28.7] prior to therapy with trabectedin in comparison to 16.1 [range: 6.1–32.4] following treatment. We compared the average SUV values with the clinical response data according to RECIST criteria using conventional MRI and/or CT. One patient achieving PR showed a significant decrease of the SUV (6.7 > 3.5, decrease of 48%) and the SUV_max_ (13.5 > 6.1, decrease of 54%) indicating tumour response to treatment; the patient further continued treatment with trabectedin. In the literature, a cutoff value of 40% reduction from baseline has been chosen to differentiate responders [25]. Three patients with SD after one (n = 2) or three (n = 1) cycles of trabectedin demonstrated no major change in SUV indicating tumour stabilization; all patients continued treatment with trabectedin. Two patients with PD under treatment with trabectedin also showed stabilization of the SUV; nevertheless, treatment with trabectedin was stopped due to RECIST criteria. Two patients received only one cycle of trabectedin and died within one month after start of therapy due to PD and therefore were not evaluable regarding sequential PET examinations. One patient received two cycles of trabectedin and showed documented PD; no PET follow-up examination was performed due to refusal of the patient. Hence, a significant decrease (> 40%) of the SUV of the sequential PET examinations could be demonstrated for one responding patient (PR), whereas patients with SD or even PD showed stabilization of the SUV. There was no patient in this series demonstrating an increase of the SUV. The PET examinations seem to confirm what we know about the characteristics of trabectedin; it has a remarkable ability to slow the growth and stabilize the tumour, which is reflected by our results regarding SUV values. Moreover, we could show that sequential PET imaging with a second PET scan after treatment with trabectedin may be used to determine whether STS patients will benefit from therapy or not.

### Example

**Figure 1:**
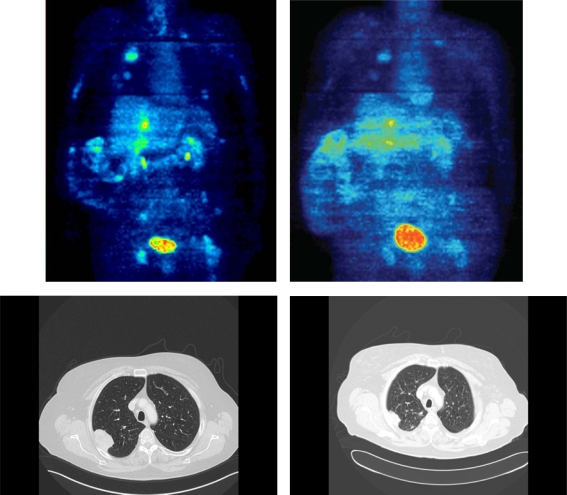
Case of a 62 year old female with a leiomyosarcoma, grade III, of the vena cava with lung and liver metastases. FDG PET showed a decrease of the average SUV from 6.7 and of the SUV_max_ from 13.5 (*upper left image*) to 3.5 and 6.1 (*upper right image*), respectively, as well as a decline of the FDG uptake in all evaluable lesions (mSUV PET images, colour-scales are directly comparable (0–10 SUV)). As an example, the corresponding CT scan showed the reduction in size of one reference lesion in the lung demonstrating a PR according to RECIST criteria (*lower left and right images*). Additionally, liver metastases demonstrated a partial remission (not shown).

In order to demonstrate that PET imaging may provide additional information compared to conventional MRI and/or CT, we describe a case from one of our patients (Figure 1): A 62 year old female with a leiomyosarcoma, grade III, of the vena cava with lung and liver metastases. The FDG PET prior therapy with trabectedin showed an average SUV of 6.7 and a SUV_max_ of 13.5 (*upper left image*). After three cycles of treatment with trabectedin, the FDG PET demonstrated a decrease of the average SUV to 3.5 and of the SUV_max_ to 6.1 (*upper right image*) and a decrease of the FDG uptake in all other evaluable lesions. The corresponding conventional CT documented PR according to RECIST (*lower left and right images*). This case underlines the common finding that the structure of the tumour may change reflected by a decline of the average and the maximum SUV caused by a change of the vessel density, blood circulation or necrosis even though the tumour size itself remains rather stable.

### Discussion

There have been different implications for the use of PET in STS. It has been studied to predict the malignant potential and grade of tumours, to stage the malignant disease, to monitor tumour response to chemotherapy, to predict clinical benefit from chemotherapy, and to detect tumour recurrence after primary therapy [26]. However, most of the studies have analysed only a small number of patients and have used different imaging protocols and evaluation procedures. Therefore, comparison of the different studies and analysis seems to be difficult. Nevertheless, there is no doubt that FDG PET is useful in the clinical management of bone and soft tissue sarcoma patients [17].

During the last decade change in tumour size to cytotoxic treatment has been the parameter to predict the therapeutic benefit for the patients. However, changes in tumour size in soft tissue sarcomas measured with CT and/or MRI were not correlated consistently with patients’ outcomes. Especially for gastrointestinal stromal tumours (GIST), this finding has already been well documented. A study of FDG PET in imatinib treated GIST showed that patients with normalization of tumour SUV within the first month of treatment have significantly longer time to progression of disease and better overall survival than those patients with increased FDG accumulation [27]. FDG PET appears to be more useful than CT/MRI imaging in GIST to predict response to therapy. Moreover, there is even doubt if RECIST criteria adequately describe the remission status to chemotherapy or to other targeted agents.

To our knowledge, the present paper describes the first small series of heavily pre-treated patients under therapy with trabectedin being monitored using sequential PET imaging. The purpose of the study was to describe certain PET characteristics of trabectedin and to identify patients who are likely to benefit from treatment. In our own study with high grade STS patients receiving neoadjuvant chemotherapy, we could demonstrate that FDG PET may be used as a tool to predict patients’ therapy outcome while differentiating between responders and non-responders after a limited drug exposure [28]. There is only one phase I study using FDG PET to monitor patients receiving trabectedin. Although there were no objective responses in that study, clear evidence of anti-tumour activity was observed in one patient with epithelioid mesothelioma as confirmed by a 40% reduction in FDG uptake after two cycles [21]. In our population of heavily pre-treated patients, we demonstrated a more than 40% decline of the SUV in our sequential PET examination for one responding patient (PR), whereas all patients with SD or even PD showed stabilization of the SUV. There was no patient demonstrating a SUV increase. However, the present data could not add any additional information to conventional imaging methods using CT and/or MRI. PET scan should be most useful in patients with SD; however, there was no change in SUV in this group of patients. Of course, these limitations of the study could be due to the low number of evaluated patients or to the short follow-up time of the present data.

## 4. Conclusions

Taken together, FDG PET will certainly play an increasingly important prognostic and predictive role in the management of sarcomas. It could be used to assess the aggressiveness of the tumour in order to make early clinical decisions whether treatment is useful for the patient or not [28]. Our present data suggest that the characteristics of trabectedin are reflected by PET examinations. Trabectedin shows a remarkable ability to slow the growth and stabilize the tumour indicated by stabilization or even decrease of the SUV values. Furthermore, PET imaging may be used in order to predict response to therapy early in the course of treatment for cytotoxic chemotherapy as well as targeted agents such as kinase inhibitors or trabectedin. However, more data have to be evaluated. FDG PET imaging may complement radiological tomography and histological grading, thus improving the assessment of STS and implying an influence on therapeutic decisions in the future.

## Figures and Tables

**Table 1:** Patients’ characteristics (n = 9).

*Gender*		
	Female	6
	Male	3
*Age*		
	Median (years)	52 [range: 26–73]
*Histology*		
	Synovial sarcoma	2
	Leiomyosarcoma	7
*Grading*		
	II	2
	III	7
*Tumour site at initial diagnosis*		
	None	1
	Extremities	3
	Trunk	5
*Metastatic sites*		
	Lung	8
	Liver	3
	Lymph nodes	3
	Soft tissue	2
*Line of Treatment*		
	2^nd^ line	4
	3^rd^ line or more	5

## References

[1] Pisters P Pollock RE Staging and Prognosis American Cancer Society Atlas of Clinical Oncology: Soft Tissue Sar comas BC Decker, Inc Hamilton, ON, Canada 2002 80 88

[2] Standard-Options-Recommandations Sarcome des T issus Mous et Ostéosarcomes Arnette Blackwell Paris, France 1995 1 1 113

[3] Verweij J Mouridsen HT Nielsen OS Woll PJ Somers R van Oosterom AT van Glabbeke M Tursz T The present state of the art in chemotherapy for soft tissue sarcomas in adults: The EORTC point of view Crit Rev Oncol Hematol 1995 20 193 201 8748009 10.1016/1040-8428(94)00146-K

[4] Frustaci S de Paoli A Bidoli E Ifosfamide in the adjuvant therapy of soft tissue sarcomas Oncology 2003 65 80 84 14586155 10.1159/000073366

[5] Grobmyer SR Maki RG Demetri GD Mazumdar M Riedel E Brennan MF Singer S Neo-adjuvant chemotherapy for primary high-grade extremity soft tissue sarcoma Ann Oncol 2004 15 1667 1672 15520069 10.1093/annonc/mdh431

[6] Pisters PW Patel SR Varma DG Cheng SC Chen NP Nguyen HT Feig BW Pollack A Pollock RE Benjamin RS Preoperative chemotherapy for stage IIIB extremity soft tissue sarcoma: long-term results from a single institution J Clin Oncol 1997 15 3481 3487 9396401 10.1200/JCO.1997.15.12.3481

[7] Wendtner CM Abdel-Rahman S Krych M Baumert J Lindner LH Baur A Hiddemann W Issels RD Response to neoadjuvant chemotherapy combined with regional hyperthermia predicts long-term survival for adult patients with retroperitoneal and visceral high-risk soft tissue sarcomas J Clin Oncol 2002 20 3156 3164 12118030 10.1200/JCO.2002.07.146

[8] Strauss LG Conti PS The applications of PET in clinical oncology J Nucl Med 1991 32 623 648 2013803

[9] Schulte M Brecht-Krauss D Heymer B Guhlmann A Hartwig G Sarkar MR Diederichs CG Schultheiss M Kotzerke J Reske SN Fluorodeoxyglucose positron emission tomography of soft tissue tumors: is a non-invasive determination of biological activity possible? Eur J Nucl Med 1999 26 599 605 10369945 10.1007/s002590050427

[10] Schwarzbach M Dimitrakopoulou-Strauss A Willeke F Hinz U Strauss LG Zhang YM Mechtersheimer G Attigah N Lehnert T Herfarth C Clinical value of [^18^F]fluorodeoxyglucose positron emission tomography imaging in soft tissue sarcomas Ann Surg 2000 231 380 386 10714631 10.1097/00000658-200003000-00011 PMC1421009

[11] Eary JF Mankoff DA Tumor metabolic rates in sarcoma using FDG PET J Nucl Med 1998 39 250 254 9476930

[12] Schelling M Avril N Nahrig J Kuhn W Romer W Sattler D Werner M Dose J Janicke F Graeff H Schwaiger M Positron emission tomography using [^18^F]fluorodeoxyglucose for monitoring primary chemotherapy in breast cancer J Clin Oncol 2000 18 1689 1695 10764429 10.1200/JCO.2000.18.8.1689

[13] Gretschel S Moesta KT Hünerbein M Lange T Gebauer B Stroszczinsk C Bembenek A Schlag PM New concepts of staging in gastrointestinal tumors as a basis of diagnosis and multimodal therapy Onkologie 2004 27 23 30 15007245 10.1159/000075603

[14] Dimitrakopoulou-Strauss A Strauss LG Burger C Ruhl A Irngartinger G Stremmel W Rudi J Prognostic aspects of ^18^F-FDG PET kinetics in patients with metastatic colorectal carcinoma receiving FOLFOX chemotherapy J Nucl Med 2004 45 1480 1487 15347714

[15] Stroobants S Goeminne J Seegers M Dimitrijevic S Dupont P Nuyts J Martens M van den Borne B Cole P Sciot R Dumez H Silberman S Mortelmans L van Oosterom A ^18^FDG-Positron emission tomography for the early prediction of response in advanced soft tissue sarcoma treated with imatinib mesylate (Glivec) Eur J Cancer 2003 39 2012 2020 12957455 10.1016/s0959-8049(03)00073-x

[16] Schuetze SM Rubin BP Vernon C Hawkins DS Bruckner JD Conrad EU 3rd Eary JF Use of positron emission tomography in localized extremity soft tissue sarcoma treated with neoadjuvant chemotherapy Cancer 2005 103 339 348 15578712 10.1002/cncr.20769

[17] Schuetze SM Utility of positron emission tomography in sarcomas Curr Opin Oncol 2006 18 369 373 16721133 10.1097/01.cco.0000228744.49294.12

[18] Verweij J Ecteinascidin-743 (ET-743): Early test or effective treatment in soft tissue sarcomas? J Clin Oncol 2005 24 5420 5423 16109999 10.1200/JCO.2005.04.905

[19] Fayette J Coquard IR Alberti L Ranchère D Boyle H Blay JY ET-743: A novel agent with activity in soft-tissue sarcomas Curr Opin Oncol 2006 18 347 353 16721129 10.1097/01.cco.0000228740.70379.3f

[20] Grosso F Forni C Frapolli R Greco A Gronchi A Jimeno J Mantovani R D'Incalci M Pilotti S Casali PG Sensitivity of myxoid-round cell liposarcoma (MRCL) to trabectedin (T) may be related to a direct effect on the fusion transcript Proc Am Soc Clin Oncol 2007 25 18S (abstract 10000)

[21] Ryan DP Supko JG Eder JP Seiden MV Demetri G Lynch TJ Fischman AJ Davis J Jimeno J Clark JW Phase I and pharmacokinetic study of ecteinascidin 743 administerd as a 72-hour continuous intravenous infusion in patients with solid malignancies Clin Cancer Res 2001 7 231 242 11234874

[22] Trojani M Contesso G Coindre JM Rouesse J Bui NB de Mascarel A Goussot JF David M Bonichon F Lagarde C Soft-tissue sarcomas of adults; study of pathological prognostic variables and definition of a histopathological grading system Int J Cancer 1984 33 37 42 6693192 10.1002/ijc.2910330108

[23] Schwarzbach MH Hinz U Dimitrakopoulou-Strauss A Willeke F Cardona S Mechtersheimer G Lehnert T Strauss LG Herfarth C Buchler MW Prognostic significance of preoperative [18-F] fluorodeoxyglucose (FDG) positron emission tomography (PET) imaging in patients with resectable soft tissue sarcomas Ann Surg 2005 241 286 294 15650639 10.1097/01.sla.0000152663.61348.6f PMC1356914

[24] Burger C Buck A Requirements and implementation of a flexible kinetic modelling tool J Nucl Med 1997 38 1818 1823 9374364

[25] Cheng EY Froelich JW Manivel JC Weigel BJ Skubitz KM Correlation of FDG-PET with histologic response after neoadjuvant chemotherapy for soft tissue sarcomas Proc Am Soc Clin Oncol 2009 25 18S (abstract 10583)

[26] Dimitrakopoulou-Strauss A Strauss LG Schwarzbach M Burger C Heichel T Willeke F Mechtersheimer G Lehnert T Dynamic PET ^18^F-FDG studies in patients with primary and recurrent soft tissue sarcomas: impact on diagnosis and correlation with grading J Nucl Med 2001 42 713 720 11337565

[27] Jager PL Gietema JA van der Graaf WT Imatinib mesylate for the treatment of gastrointestinal stromal tumours: best monitored with FDG PET Nucl Med Commun 2004 25 433 438 15100500 10.1097/00006231-200405000-00002

[28] Kasper B Dietrich S Dimitrakopoulou-Strauss A Strauss LG Haberkorn U Ho AD Egerer G Early prediction of therapy outcome in patients with high risk soft tissue sarcoma using positron emission tomography Onkologie 2008 31 107 112 18322413 10.1159/000113795

